# Predictors of Number of Healthcare Professionals Consulted by Individuals with Mental Disorders or High Psychological Distress

**DOI:** 10.3390/ijerph16173010

**Published:** 2019-08-21

**Authors:** Béatrice Simo, Jean Caron, Jean-Marie Bamvita, Guy Grenier, Marie-Josée Fleury

**Affiliations:** 1 École de santé publique, Université de Montréal, 7101 av. du Parc, Montréal, QC H3X1X9, Canada; 2 Research Centre, Douglas MH University Institute, 6875 LaSalle Blvd, Montreal, QC H4H 1R3, Canada; 3 Department of Psychiatry, McGill University, 1033, Pine Avenue West, Montreal, QC H3A 1A1, Canada

**Keywords:** predictors, number of diversified healthcare professionals consulted, needs, mental disorders, high psychological distress

## Abstract

This study assesses the contribution of predisposing, enabling, and needs factors and related variables that predicted the number of healthcare professionals consulted for mental health reasons among 746 individuals with mental disorders and high psychological distress. The data were drawn from the third (T3) and fourth data collection periods (T4) of a longitudinal study conducted in a Quebec/Canada epidemiological catchment area. Hierarchical linear regression was performed on the number of types of healthcare professionals consulted in the 12 months prior to T4. Predictors were identified at T3, classified as predisposing, enabling, and needs factors (i.e., clinical and related variables) according to the Andersen Behavioral Model. Three needs factors were associated with the number of types of healthcare professionals consulted: Post-traumatic stress disorder, stressful events, and marginally suicide ideation. Three enabling factors: Having a family physician, previous use of mental health services, and employment status were also related to the dependent variable. Poor self-perception of mental health status was the only predisposing factor retained. While needs factors were the main predictors of the number of types of healthcare professionals consulted, enabling factors may reduce the influence of needs factors, by the deployment of various strategies that facilitate continuous and appropriate care.

## 1. Introduction

Mental disorders (MD) represent a challenge to the healthcare system due to their high prevalence, costs, and burden of disability, while negatively impacting quality of life for affected individuals and families [1,2]. Recurring MD and physical comorbidity are also common [3,4], leading many individuals to seek extensive and diversified care [5]. According to previous studies, as many as 70% of individuals affected by MD who use services consult professionals in primary care settings for prevalent mental health (MH) conditions such as anxiety, mood, and substance use disorders [6,7], while approximately 36% of individuals with MD seek care from general practitioners and MH specialists [5]. General practitioners at the entry point to primary care are largely responsible for care co-ordination and patterns of service use. Patients perceive help received from general practitioners in combination with MH specialists as highly effective [8]. For instance, psychotherapy provided by a psychologist is commonly recommended for the treatment of common MD (e.g., depression, anxiety), with medication provided by a general practitioner [9]. Case managers, often nurses or social workers, are involved with patient follow-up in many best-practice models. They oversee medication adherence and healthy living practices as well as crisis management [10,11]. General practitioners may also request consultations with psychiatrists for assistance in establishing diagnoses or prescribing treatments for MD, particularly in complex cases or recurrent illness [12].

Collaborative care models have been identified as particularly successful in delivering integrated medical and MH care [13,14], with demonstrated effectiveness for improving access to care, patient outcomes and satisfaction, and for controlling costs [15]. Effective management of MH patients involves close collaboration between general practitioners and MH professionals working in primary care settings (e.g., nurses, psychologists, social workers) and with psychiatrists from specialized care settings [9,16]. In publicly funded health systems, as in Canada, individuals with MH problems are generally seen first in primary care settings by general practitioners or family physicians, then referred to MH specialists or other healthcare professionals as needed. In Quebec, collaborative care models in MH were vigorously promoted in the context of the 2005 MH Action Plan and reinforced in the new MH Action Plan of 2015. In this context of MH system transformation, it may be useful to identify variables that predict the number of types of healthcare professionals consulted for MH reasons. While studies abound on the frequency of MH service use, and emergency department use in particular [17,18,19], relatively few studies have addressed MH service use with a focus on the number of different types of healthcare professionals consulted [5,20].

Considerable research on MH service utilization has been guided by the Andersen Behavioral Model [21]. This model represents the dominant framework for understanding treatment seeking and identifying predictors of service utilization [22]. According to this model, variables that influence healthcare service utilization, including MH services, may be grouped according to three types of factors: (1) Predisposing factors, including individual socio-demographic characteristics that predate the onset of illness (e.g., age, civil status), and health beliefs (e.g., attitudes, values, and knowledge related to personal health and health services) that may inform perceptions of need and future service use [21]; (2) enabling factors, or resources that facilitate service access (e.g., having a family physician, previous use of MH services); and (3) needs factors, including clinical variables such as diagnoses and other needs variables such as those related to functional disability [21,22]. The Andersen Behavioral Model has the great advantage of including a broad range of variables that may facilitate or hinder healthcare service utilization and specifying the relative influence of these factors. The results of most studies suggest that needs factors account for most health service use [21].

Concerning predisposing factors, previous research has found a positive association between age and use of both psychiatrists and general medical professionals [23]. A Canadian study with a community-based adult sample found that women were more likely than men to consult general practitioners and psychologists; or general practitioners and other health professionals (e.g., nurse, social worker, counsellor) with the exception of psychiatrists [24]. Concerning enabling factors, individuals who enjoyed excellent relationships with their neighbors, consulted relatively higher numbers of types of professionals for MH problems [5]. Regarding needs factors, individuals with previously diagnosed depression were more likely to seek help from both a general practitioner and psychiatrist; while having more than one MD was associated with more types of professionals consulted [5]. Psychological distress stood out in one study as the best single predictor for all types of healthcare consultations (general practitioners, MH specialists, other healthcare professionals) [25]. Participants with unmet MH needs also were more likely to seek help from general practitioners, psychologists, or other professionals (e.g., psychiatrists, nurses, social workers) [26].

Previous studies on MH service use have mainly focused on samples of individuals with MD only, even though an appreciable proportion of MH service users have subthreshold MD and needs that may differ from those of individuals who meet the criteria for a diagnosed MD [7,27,28]. In one systematic review that analyzed the results of 18 epidemiological studies including 48,214 participants, the prevalence of subthreshold generalized anxiety disorder (GAD) was twice that of diagnosed GAD; however, people with subthreshold GAD had more persistent symptoms than those with anxiety disorders, which caused suffering, psychosocial, and work-related impairment. They also were at a greater risk for developing threshold GAD and other anxiety, mood, and substance use disorders [29]. In addition, having an undiagnosed MD was a significant predictor of lifetime hospitalization, lifetime MD without current symptoms, suicide attempts, high psychological distress, and other significant disability [27].

In order to form a better picture of individuals in need of MH services, it may be useful to investigate MH service utilization among individuals without a diagnosis, but who are at high risk for MD [30]. Accordingly, this study focused on both individuals with a diagnosed MD and those with high psychological distress as measured by: (1) Symptoms similar to those associated with common MD including depression and anxiety [31]; (2) conditions likely to evolve into MD if left untreated; and (3) conditions associated with high levels of functional impairment [32]. MH service utilization studies that include participants with high psychological distress at risk for developing MD, as well as diagnosed individuals, also conform to MH policies encouraging early intervention [33,34]. Thus, using the Andersen model, this study aims: (1) To assess the relative contribution of predisposing, enabling, and needs factors on the number of types of healthcare professionals consulted for MH reasons and (2) to identify predictors of a greater diversity of healthcare professionals consulted in a sample of both individuals with diagnosed MD and those with high psychological distress. As needs factors were mainly associated with health service use in previous studies, we hypothesized that individuals with more serious needs would seek help from a greater variety of MH professionals.

## 2. Method

### 2.1. Study Design and Setting

This study is based on a subsample from a longitudinal population-based cohort study, conducted in an epidemiological catchment area in Southwest Montreal, Quebec (Canada), with a population of 269,720 distributed among six neighborhoods. Data for this specific study (the subsample) included the two last data collections from the full longitudinal study (T3, T4). The area includes a major psychiatric hospital offering specialized services for serious and complex MD, and two community health and social service centers that deliver primary MH care. Numerous private clinics employ general practitioners and psychologists, while community organizations offer supportive services such as crisis centers, day centers, and self-help groups for individuals with MH problems and their families.

### 2.2. Selection Criteria and Study Population

Study participants had to be between 15 and 65 years of age and live within the catchment area. A geographically representative sample with socio-economic and other characteristics proportional to those of the general population was sought. A random sample of 3408 home addresses was initially selected for recruitment purposes. In order to improve the recruitment process, this initial list was extended to include a range of 14 neighboring addresses for door-to-door recruitment [35]. Interviews were scheduled with individuals who agreed to participate either at home or at another designated location. Trained interviewers administered questionnaires to participants. The duration of interviews ranged from 90 to 135 min depending on whether or not participants had positive signs of MD and/or used MH services. Each participant signed a consent form prior to the interview. The anonymity of participants was guaranteed, and data were kept in a place accessible only to project researchers. Telephone contacts were made every six months encouraging participants to participate in the subsequent data collection periods. The ethics review board of a MH institute approved the multi-site protocol. 

**Figure 1:**
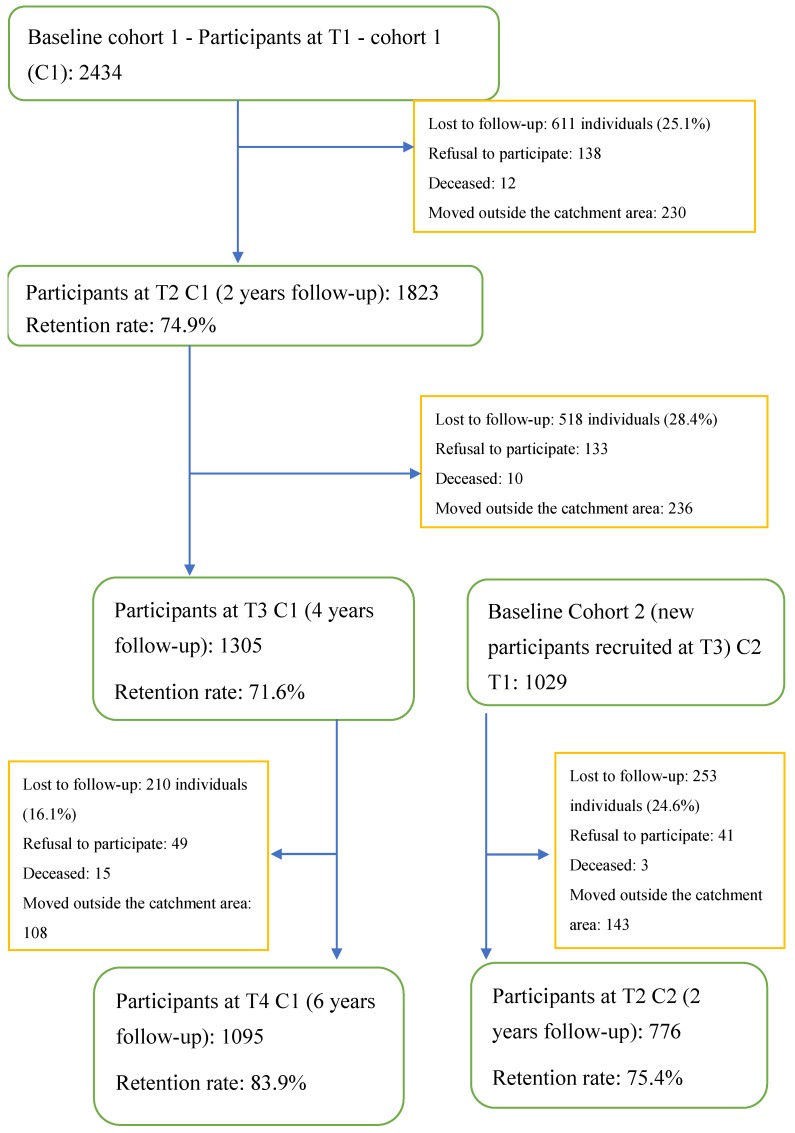
Recruitment flowchart from Time 1 (T1) to T4 data collection periods. Data collection for the present study included: T3 (independent variables) and T4 (dependent variable).

The first data collection period (T1: June 2007–December 2008) included N = 2434 study participants. Three additional data collection periods followed at two-year intervals (T2: June 2009–December 2010; T3: June 2011–December 2012; T4: June 2013–December 2015); they are summarized in Figure 1. The response rate for this longitudinal cohort was: 74.9% at T2, 72% at T3, and 80% at T4. These response rates were slightly higher than those in comparable longitudinal epidemiological studies (69% to ~76% for other two to five year studies) [36,37]. Most dropout between data collection periods involved lower participation by younger or materially deprived individuals and those diagnosed with substance use disorders. Further details on selection criteria and sample characteristics have been published elsewhere [5,35]. At the fourth data collection period, the mean age of participants (N = 1871) was 44.72 years (SD: 13.86); 60% were female, and a small majority lived alone (52%). 

### 2.3. Conceptual Framework, Variables, and Instruments

**Figure 2:**
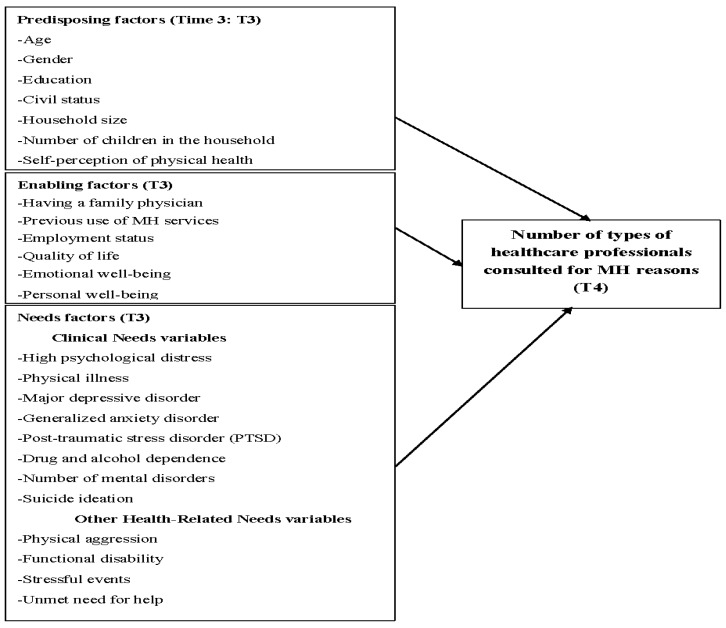
Conceptual framework: (N = 746 individuals with mental disorders (MD) or high psychological distress).

The dependent variable, “number of types of healthcare professionals consulted for MH reasons”, was measured for each participant at T4. Professional consultation was defined as contact with any healthcare professional for MH reasons in the 12 months prior to the interview: General practitioner, psychiatrist, psychologist, nurse, social worker, etc. Independent variables (measured at T3) were integrated based on the literature related to predictors of MH service use [21,22,38] and were classified according to the Andersen model. Predisposing variables included: Age, gender, education, civil status, household size, number of children in the household, and health beliefs such as self-perception of mental health and self-perception of physical health, as well as satisfaction with health services. Enabling variables included: Having a family physician, previous use of MH services (consultations with any healthcare professionals or community service organizations such as self-help groups), and employment status. As MH service use is known to have a strong influence on outcomes such as quality of life, emotional well-being, and personal well-being [21], these variables were also included as enabling factors. Needs factors were categorized as: (a) Clinical needs, i.e., diagnoses (major depressive disorder, generalized anxiety disorder, post-traumatic stress disorder (PTSD), drug and alcohol dependence, as well as high psychological distress, physical illnesses, suicide ideation and (b) other health-related needs, including stressful events, unmet need for help, physical aggression, and functional disability. MH diagnoses with low prevalence such as schizophrenia or personality disorders were not included in the study, as is often the case in population-based studies [39,40]. The analytical framework is presented in Figure 2. Table 1 displays the standardized instruments used to measure the variables or dimensions identified above, including sub-dimensions measured and numbers of items, ranges for each score, interpretation of the score, and where possible the Cronbach alpha. These standardized instruments were selected as most of them were used previously in the Canadian Health Survey—Mental health and well-being (CCHS) 1.2 [41], which is however not a longitudinal population study.

### 2.4. Analyses

Univariate, bivariate, and multivariate analyses were performed. Missing values were also treated by imputation technics (however, fewer than 5% of variables had missing values). Univariate analyses consisted of frequency distributions for categorical variables and central tendency measures (mean values and standard deviations) for continuous variables. A bivariate linear regression analysis was conducted for each independent variable to determine which ones were associated with a higher number of different types of healthcare professionals consulted for MH reasons. Significant independent variables in the bivariate analyses (*p* < 0.10) were used to build the hierarchical linear regression model, with the alpha value set at *p* < 0.05 to determine which factors in the model were significant. Following the study hypothesis, clinical needs variables were entered into the model first, followed by other needs variables, then predisposing and enabling factors. Needs factors were divided into “clinical” needs factors (e.g., diagnoses) and “other” needs factors (e.g., functional disability) to highlight the differences between them in terms of impact on service utilization. For each block of variables, the goodness-of-fit was determined with the Hosmer–Lemeshow test and the variance explained using the Nagelkerke R^2^. 

## 3. Results and Discussion

### 3.1. Results

Out of the large epidemiological catchment study, 1871 participants were selected from T4 for the present study. Within this group, 746 had either a diagnosed MD (N = 201; 27%) or high psychological distress (N = 713; 96%). Table 2 shows the main characteristics of those participants at T3, as well as bivariate associations with the number of healthcare professionals visited at T4. Regarding predisposing factors, mean age was 44 years (Table 2). Participants consisted of nearly twice as many women as men (62% vs. 38%). Most lived alone (63%). Their self-perception of physical health (2.8 on 5; SD: 1.1) and of MH (2.9 on 5; SD: 1.0) were average, as well as their satisfaction with MH services (51.7 on 100; SD: 13.8). Regarding enabling factors, over two thirds (69%) had a family physician. Over the previous 12 months, 29% (N = 219) had visited a healthcare professional for MH reasons. Of the total sample, participants most frequently consulted general practitioners (15%, N = 112), followed by psychologists at 14% (N = 104) and psychiatrists at 8% (N = 60). Sixty-two percent of those who visited a healthcare professional (N = 136/219) consulted a single healthcare professional, usually a general practitioner (25%, N = 34/136); while 26% (N = 56/219) consulted two healthcare professionals, usually a general practitioner and a psychologist (57%, N = 32/56), and 11% (N = 25/217) at least three healthcare professionals, usually a general practitioner, psychologist, and psychiatrist. Only 19% had held a job during the previous year. Regarding needs factors, major depression was most prevalent (17%), and 9% experienced suicide ideation. The psychological distress score was relatively high (13.8; SD: 5.3). The mean number of stressful events per participant was 3.8 (SD: 2.4). Physical illnesses were also prevalent among participants (64%). Independent variables significantly associated with the dependent variable, number of types of healthcare professionals consulted for MH reasons, in the bivariate analyses (Table 2) were used to build the hierarchical linear regression model. 

The hierarchical linear regression model is presented in Table 3 and includes four blocks: Clinical needs, other health-related needs (needs factors), predisposing, and enabling factors. The first factor, clinical needs, yielded five positively associated variables: Psychological distress, major depressive disorder, generalized anxiety disorder, PTSD, and suicide ideation. These variables remained significantly associated with the dependent variable after the introduction of the second block, other health-related needs factors, with the exception of psychological distress, and two new variables were added: Stressful events and functional disability, which were both positively associated with the dependent variable. After introduction of the third block, predisposing factors, one variable, self-perception of MH, was negatively associated with the dependent variable, meaning that those who perceived their MH as excellent were less likely to visit a greater variety of healthcare professionals for MH reasons. Regarding the fourth block, enabling factors, three variables, previous use of services for MH reasons, having a family physician, and employment status were positively associated with the dependent variable, in addition to the four previously entered variables: PTSD, stressful events, self-perception of MH, and (marginally) suicide ideation. The total variance explained by the four model was 21%: 11% for clinical needs and 2% for other health-related needs, 2% for predisposing factors, and 6% for enabling factors, respectively to the final model. The goodness-of-fit was acceptable.

### 3.2. Discussion

Based on this catchment area study, slightly less than one-third of the sample reported consulting healthcare professionals for MH reasons in the previous year. This result falls within the low range of consultations (from 5% in low income to 44% in high income countries) observed in previous population studies [22,55,56], which may be explained by the inclusion of individuals with high psychological distress in our sample of diagnosed individuals with MD. Among those who consulted a single healthcare professional, general practitioners and psychologists were equally favored, followed by psychiatrists. This order in the choice of healthcare professionals is consistent with previous Quebec epidemiological studies [24,57]. The finding that fewer than four in ten individuals had used at least two healthcare professionals makes sense, as psychotherapy and treatment with medication are generally recommended in combination with the management of MH conditions in step-care models [16,58]. The finding that a minority (about 10%) required consultations with psychiatrists corresponded to the rate of more complex, recurrent, or serious MD in the sample [59]. It is well known that general practitioners have difficulty treating such complex cases in primary care settings [60].

The hierarchical regression model revealed that needs factors predicted the highest number of types of healthcare professionals consulted for MH reasons, followed by enabling factors; whereas the contribution of predisposing factors was marginal. These findings confirm our hypothesis that individuals with complex or serious needs would be more inclined to consult a greater number of different types of healthcare professionals. However, it should be noted that there were equivalent numbers of significant needs factors (PTSD, stressful events, and (marginally) suicide ideation) and enabling factors (previous use of MH services, having a family physician, and employment status) in the final model; whereas only one predisposing factor (self-perception of MH) remained significant. Interestingly, three needs factors (major depressive disorder, generalized anxiety disorder, and functional disability) ceased to be associated with the dependent variable after the block of enabling factors was introduced into the model, suggesting that the presence of enabling factors substantially reduced the influence of these needs factors on number of different types of healthcare professionals consulted for MH reasons. 

As predicted in this study, and reported previously [24,61], we found that the presence of a diagnosed MD was associated with the likelihood of consulting a greater number of healthcare professionals. Specifically, two clinical needs variables (PTSD and (marginally) suicide ideation) were associated with the dependent variable in the final model. A Canadian study found that individuals with PTSD usually experience chronic symptoms causing functional impairment and high rates of comorbidity [62]. Another study found an association between PTSD and substantial reduction in quality of life affecting general health, energy levels, emotional and physical well-being, as well as social functioning [63]. Reports indicate that individuals with suicide ideation are also more likely to seek treatment from both a general practitioner and MH specialist [64]. This may reflect a perceived need for MH care on the part of individuals with suicidal thoughts and behaviors [65]. Among individuals with suicide ideation, perceived need for MH care has been associated with increased likelihood of service use [65]. A study on MH care for adults with suicide ideation [66] found that perceived need for care predicted nearly four times the likelihood of receiving treatment. ‘Stressful life events’ was the only other health-related needs variable associated with the number of types of healthcare professionals consulted for MH reasons. There is considerable evidence that individuals who have experienced an above average number of negative life events are more likely to experience psychological distress [67]; they also have greater odds of developing PTSD [68] or other MD [69,70,71] and are more likely to seek MH services [68,72].

Three enabling factors predicted consultations with a greater number of different types of healthcare professionals for MH reasons: Previous use of MH services, employment status, and having a family physician. Due to the chronic nature of certain MH conditions, episodes of relapse or recurrence [4] may require adherence to treatment over a longer period for achieving better outcomes. Past research has also found that prior treatment history for MD was associated with more positive attitudes toward medical interventions [73] and help-seeking from various providers [74]. Knowledge and experience with services are recognized as facilitators in accessing care [75]. Having a family physician also provides an entry point to the healthcare system: Family physicians diagnose and treat patients who present with MH symptoms, making referrals of more severe or complex cases to specialized MH professionals [76,77]. By contrast, not having a family physician may result in unmet needs for care [78] or delayed access to MH care, especially among individuals unaware of their MH symptoms and the available resources [75]. This finding underscores the importance of integrating MH into primary care, improving continuity of care by making family physicians universally available, and increasing their training in the screening and treatment of MH problems, and better supporting them through collaborative care models. Employment status was another predictor of the number of different types of healthcare professionals consulted for MH reasons. Quite possibly, employed individuals have greater access to certain healthcare professionals, including private psychologists [5]. Moreover, employee assistance programs may offer workplace educational support, counseling, confidential screening, and assessment [79]. While unemployed individuals may feel depressed or have a greater need for MH care [80], those who occupy positions with high levels of job insecurity or negative stress may be at even higher risk of MH problems. For instance, one study [81] reported that employed individuals with perceived job insecurity were more likely to have poor MH status and greater need for care than unemployed individuals; they also made greater use of MH services [81].

Finally, one predisposing factor, self-perception of MH, negatively predicted the number of different types of healthcare professionals consulted for MH reasons. This corresponds to previous findings suggesting that patients who had a worse self-perception of their MH were more likely to seek help from general practitioners and MH specialists rather than relying on a single healthcare professional [82,83]. Another study [24] found that individuals who rated their MH worse were more likely to seek help from either a general practitioner and psychologist, or a general practitioner and psychiatrist. This finding underscores the relevance of individual perceptions on the need for care in the absence of a MD and MH service utilization.

### 3.3. Limitations

The findings of this study should be considered in light of certain limitations. First, the frequency of consultations with each healthcare professional was not taken into account. As such, continuity and appropriateness of care were not considered, which are key dimensions impacting quality of care and patient recovery. Second, the diversity of types of healthcare professionals consulted did not take into account the existence of actual collaboration among them, as required in collaborative care models. Third, some pertinent predictors of consultations with different types of healthcare professionals for MH reasons that may influence service utilization, including lifetime disorders, symptoms severity, suicide attempt [27], ethnicity, or religion [84] were not measured in this study. Fourth, populations younger than 15 and older than 65 were excluded from the study, as MH health service use patterns for these age groups are very distinct. Fifth, the study did not cover the full spectrum of MD, as some disorders assessed in the third data collection period were not assessed in the fourth data collection. Serious MD with very low population prevalence, such as schizophrenia and personality disorders, were also excluded. Six, the final study sample (at T4) was less representative of the population (i.e., in terms of youth, individuals with high material deprivation, and substance use disorders). Finally, the total variance (21%) explained by our model was relatively low.

## 4. Conclusions 

This study was innovative in assessing predictors of diversity in the number of types of healthcare professional consultations for MH reasons among individuals, not only those diagnosed with MD, but mainly those affected by high psychological distress, using the Andersen Behavioral Model. This latter clientele is often overlooked by MH professionals, who focus mainly on diagnosed MD cases in their practices. Findings revealed that needs factors were most strongly associated with the number of types of healthcare professionals consulted, but that enabling factors may reduce the influence of certain needs factors, particularly those involving clinical variables. Multiple variables associated with consultations of a greater number of different types of healthcare professionals for MH reasons were identified: PTSD, stressful events, previous use of MH services, employment status, having a family physician, self-perception of MH, and (marginally) suicide ideation. The identification of specific predictors may help orient MH programs and interventions. Considering that enabling factors are easier to tackle than needs or predisposing factors, it would be important for MH managers to prioritize the development of collaborative care models to facilitate continuity and appropriateness of services for individuals who view their MH as poor. Moreover, all individuals with MH problems should have a family physician to provide an entry point to the healthcare system. Family physicians are responsible for addressing a wide range of MH needs, but also for making referrals to specialized MH services in more severe or complex cases, such as PTSD. Further extensions of employment assistance programs may facilitate better MH, as such support in the workplace may serve as an antidote to stress and related MH problems, while providing individuals with the means to consult MH specialists when needed. Finally, information on help-line services and crisis centers should be more widely disseminated, given the numbers of individuals affected by stressful events and suicide ideation.

## Figures and Tables

**Table 1:** Measurement instruments.

Variables	Instruments, References, and Psychometric Properties	Description
Predisposing factors		
Age	Canadian Community Health Survey of MH and Well-Being CCHS 1.2 [41]	Calculated from date of birth and confirmed by participants One item Numeric value
Gender	CCHS 1.2 [41]	As declared by participants Two items (male/female)
Civil status	CCHS 1.2 [41]	As declared by participants Two items (living as a couple; living alone)
Household size	CCHS 1.2 [41]	As declared by participants One item Numeric value
Number of children in the household	CCHS 1.2 [41]	As declared by participants One item Numeric value
Self-perception of physical health	CCHS 1.2 [41]	Self-perception of physical health One item Five-point Likert scale Higher = more negative
Self-perception of mental health (MH)	CCHS 1.2 [41]	Self-perception of mental health (MH) One item Five-point Likert scale Higher = more negative
Satisfaction with health services	CCHS 1.2 [41]	Measure satisfaction with health services 30 items Four-point Liker scale Higher = greater satisfaction
Enabling factors		
Having a family physician	CCHS 1.2 [41]	As declared by participants Yes/No
Previous use of MH services	CCHS 1.2 [41]	As declared by participants Yes/No
Employment status	CCHS 1.2 [41]	As declared by participants Yes/No
Personal well-being	Australian Unity Well-being Index [42] Cronbach alpha: 0.85	Measures personal satisfaction with life as a whole and in Eight sub-dimensions Nine items 10-point Likert scale Higher = more positive
Emotional well-being	MH Continuum—Short Form [43]	Measures the degree of emotional well-being defined in terms of positive affect/satisfaction with life; social well-being as described in Keyes [44] model of social well-being 14 items Seven-point Likert scale Range: 1–98 Higher = more negative
Quality of life	Satisfaction with Life Domains Scale [45]; adapted by Baker and Intagliata [46] for psychiatric patients Cronbach alpha: 0.9	Quality of life in five domains 20 items Seven-point Likert scale Range: 0–140 Higher = better quality of life
Needs factors		
Major depressive disorder	Composite International Diagnostic Interview (CIDI) and CIDI-SF [47]	Psychiatric diagnoses based on the definitions and criteria of ICD-10 ^a^ and DSM-IV ^b^. Yes/No and multiple choice
Generalized anxiety disorder	Composite International Diagnostic Interview (CIDI) and CIDI-SF [47]	Psychiatric diagnoses based on the definitions and criteria of ICD-10 and DSM-IV. Yes/No and multiple choice
Post-traumatic stress disorder (PTSD)	Composite International Diagnostic Interview (CIDI) and CIDI-SF [47]	Psychiatric diagnoses based on the definitions and criteria of ICD-10 and DSM-IV. Yes/No and multiple choice
Drug and alcohol dependence	Composite International Diagnostic Interview (CIDI) and CIDI-SF [47]	Psychiatric diagnoses based on the definitions and criteria of ICD-10 and DSM-IV Yes/No and multiple choice
Suicide ideation	Tousignant et al. [48]	Suicide ideation at life in the last 12 months Yes/No
Functional disability	12-item version of the World Health Organization Disability Assessment Schedule II (WHO-DAS-II) [49]	Measure functional disability 12 items Five-point Likert-scale Range: 0–60 Score of 45 or greater indicates substantial disability [50]
Stressful events	Lifetime Events Questionnaire [51]	Stressful events in the last 12 months related to housing, family and friends, income, love, and aggressive experiences 25 items Yes/No
High psychological distress	K-10 scale [52] Cronbach alpha: 0.93	Measure frequency of distress symptoms in the past month such as nervousness, tiredness, despair, agitation, sadness, and feeling of worthlessness 10 items Five-point Likert scale (cut-off point for determining high psychological distress = 9)
Physical illnesses	CCHS 1.2 [41]	Number of physical illness as declared by participants Yes/No
Physical aggression	Modified Observed Aggression Scale [53]	Assess 4 categories of aggressive behavior: Verbal aggression, aggression to propriety, self-inflicted aggression, physical aggression 20 items Yes/No
Unmet need for help	Perceived Need for Care Questionnaire [54]	Five items Yes/No

^a^ ICD: International classification of diseases; ^b^ DSM: Diagnostic and Statistical Manual of Mental Disorders.

**Table 2:** Participant characteristics and bivariate associations with number of types of healthcare professionals consulted for mental health (MH) reasons (N = 746).

			Frequency Distributions				Bivariate Associations with Number of Healthcare Professionals Visited at T4		
Variables at T3			Min	Max	n/Mean	%/SD	Beta	*t*	*p* Value
Predisposing factors	Age		15.00	72.00	43.71	14.04	0.011	0.289	0.773
	Gender	Female			460	61.7	1		
		Male			286	38.3	−0.063	−1.725	0.085
	Civil status	Living alone			473	63.4	1		
		Living as a couple			273	36.6	−0.106	−2.903	0.004
	Household size		0.00	13.00	2.47	1.47	−0.071	−1.950	0.052
	Self-perception of physical health in the past 12 months		1.00	5.00	2.75	1.05	−0.128	−3.529	<0.001
	Self-perception of MH		1.00	5.00	2.89	0.98	−0.287	−8.179	<0.001
	Satisfaction with health services		4.00	90.00	51.68	13.78	−0.136	−3.747	<0.001
Enabling factors	Having a family physician				515	69.0	0.130	3.568	<0.001
	Previous use of services for MH reasons				219	29.4	0.346	10.065	<0.001
	Private insurance coverage including visits to psychologist				46	6.2	0.142	3.921	<0.001
	Employment status				138	18.5	0.098	2.685	0.007
	Quality of life (QOL) (total score) ^a^		45.00	135.00	100.95	15.50	−0.199	−5.533	<0.001
	Emotional well-being ^b^		4.00	70.00	42.47	12.20	−0.159	−4.404	<0.001
	Personal well-being ^c^		2.00	90.00	58.48	13.69	−0.186	−5.168	<0.001
Needs factors	Stressful events (total score)		0.00	14.00	3.84	2.45	0.221	6.174	<0.001
	High psychological distress		0.00	35.00	13.83	5.13	0.201	5.593	<0.001
	Physical illnesses				1.36	1.47	0.079	2.169	0.030
	Unmet need for help				110	14.7	0.151	4.171	<0.001
	Major depressive episode				129	17.3	0.250	7.028	<0.001
	Generalized anxiety disorder				15	2.0	0.099	2.707	0.007
	Post-traumatic stress disorder (PTSD)				50	6.7	0.225	6.286	<0.001
	Drug and alcohol dependence				29	3.9	0.070	1.916	0.056
	Number of mental disorders		0.00	5.00	0.37	0.71	0.253	7.142	<0.001
	Physical aggression				43	5.8	0.085	2.332	0.020
	Functional disability ^d^		13.00	49.00	19.33	6.91	0.186	5.157	<0.001
	Suicide ideation				69	9.2	0.213	5.949	<0.001

^a^ Rating: 20–140; higher = better quality of life. ^b^ Rating: 1–98; higher = more negative emotional well-being. ^c^ Rating: 0–90; higher = greater personal well-being. ^d^ Rating: 0–60; higher = higher disability.

**Table 3:** Predictors of number of types of healthcare professionals consulted for mental health (MH) reasons (N = 746): Multiple linear regression model.

Variables		Block 1		Block 2		Block 3		Block 4						
		Beta	*p*	Beta	*p*	Beta	*p*	Beta	t	*p*	95.0% CI for B		Collinearity Statistics	
											LB ^a^	UB ^b^	Tolerance	VIF ^c^
Needs 1 Factors (Clinical)	(Constant)		0.128		0.278		0.012		0.761	0.447	−0.205	0.464		
	High psychological distress	0.085	0.024	0.042	0.286	−0.004	0.926	0.003	0.081	0.935	−0.012	0.013	0.676	1.480
	Major depressive disorder	0.159	<0.001	0.132	<0.001	0.102	0.007	0.055	1.476	0.140	−0.039	0.274	0.771	1.297
	Generalized anxiety disorder (GAD)	0.085	0.014	0.078	0.023	0.071	0.038	0.050	1.520	0.129	−0.084	0.662	0.981	1.020
	Post-traumatic stress disorder (PTSD)	0.146	<0.001	0.123	0.001	0.124	0.001	0.105	2.945	0.003	0.113	0.566	0.838	1.193
	Suicide ideation	0.114	0.002	0.101	0.005	0.085	0.017	0.062	1.776	0.076	−0.018	0.364	0.879	1.138
Needs 2 Factors (Other-Clinical)	Stressful events			0.126	<0.001	0.098	0.007	0.098	2.746	0.006	0.009	0.056	0.830	1.205
	Functional disability			0.085	0.028	0.080	0.036	0.050	1.355	0.176	−0.003	0.014	0.778	1.285
Predisposing	Self-perception of MH					−0.157	<0.001	−0.116	−2.983	0.003	−0.159	−0.033	0.705	1.419
Enabling Factors	Previous use of MH services							0.217	5.930	<0.001	0.258	0.513	0.797	1.255
	Having a family physician							0.112	3.358	0.001	0.082	0.311	0.954	1.048
	Employment status							0.079	2.332	0.020	0.026	0.301	0.940	1.064
Goodness of fit	F	20.038		17.340		17.450		18.543						
	*p*	<0.001		<0.001		<0.001		<0.001						
Total variance explained (Adjusted R Square)		0.113		0.133		0.150		0.206						

^a^ LB: Lower Bound; ^b^ UB: Upper Bound; ^c^ VIF: Variable inflation factor.
